# Microglia in Development and Disease

**DOI:** 10.1155/2013/736459

**Published:** 2013-08-27

**Authors:** Anirban Ghosh, Wolfgang J. Streit, Luisa Minghetti, Anirban Basu

**Affiliations:** ^1^Department of Zoology and Immunobiology Lab, Panihati Mahavidyalaya, West Bengal State University, Sodepur, Kolkata 700110, India; ^2^Department of Neuroscience, University of Florida College of Medicine and McKnight Brain Institute, Gainesville, FL 32611, USA; ^3^Experimental Neurology Section, Department of Cell Biology and Neuroscience, Istituto Superiore di Sanità, Viale Regina Elena 299, 00161 Rome-I, Italy; ^4^National Brain Research Center, Manesar, Haryana 122050, India

The first special issue on microglia was published in 1993 in *Glia* and others followed in regular, roughly ten-year intervals. Clearly, the importance of microglia has been recognized since the 1980s while the journal *Glia* has (appropriately) taken the lead in regularly reviewing microglial biology. Also there have been others. The current issue is the first one entirely devoted to microglia and perhaps not the last since research interest in this field has grown exponentially in the last two decades and continues to thrive. Why is the microglia field booming with no obvious end in sight? It is because microglia have become recognized as incredibly diverse and complex cellular players not only in virtually all kinds of brain pathology, but in many aspects of normal brain function as well. 

There is a growing realization that as the brain's immune system, these little guys might be of critical importance in terms of normal brain function, neurological disease pathogenesis (neural tissue degeneration), and restoring homeostasis after sudden destructive brain injury (neural tissue regeneration). In present years, the cells have been found to have direct and active involvement to prune and shape neuronal circuits, maintaining and monitoring overall neuronal microenvironmental health. Recent indications of their potential importance in neuropsychiatric disorders, cognitive behavior, spatial learning, and even masculinisation are now nudging microglia research into newer arenas. Further advancement in *in vivo* imaging and molecular probing techniques and innovative improvisation of different methodologies has not only opened up diverse aspects of microglia biology but also provided deeper insight into existing knowledge. However, with many advances in understanding of microglia, some basic queries and controversies regarding microglia/brain macrophages, their cellular characteristics and demarcation, origin and colonization in developing, adult and diseased brains, and their double-edged behavior and polarization in different normal and pathogenic situations still remain. This special issue represents an attempt to present a few of the many areas in which advances in microglial research have influenced our understanding of neurosciences. A total of ten articles have been assembled to discuss some of the newer as well as more traditional fields of microglia research. This special issue is a collection of articles with strong emphasis on the molecular interpretation of activities of microglia in normal and diseased brain.

The involvement of microglia in normal and pathological brain function is reflected in their molecular makeup, which presents as an incredibly diverse array of surface membrane receptors capable of receiving and processing myriads of signals created constantly in the CNS microenvironment. How such signals may regulate subsequent gene expression and microglial behavior is discussed here by J. Guedes et al. who take a close look at microglial micro-RNAs, a topic likely to receive additional attention for years to come. Another up-and-coming theme is the potential involvement of microglia in some of the more subtle brain pathologies represented by neuropsychiatric disorders where a clear pathological correlate in terms of neurons is often not apparent, and where the emerging concept of microglial involvement still remains a bit mysterious. L. R. Frick et al., who address this issue, discuss microglial involvement in neuropsychiatric disorders in the context of microglial pathology, an aspect of microglia biology that has not been addressed much in previous special issues but one that is gaining ground fast and is going to be around. The current issue also contains several contributions on persistent mainstream topics, perhaps most notably and traditionally microglial roles in neuroinflammatory disease, that is, multiple sclerosis and its various animal models, evidenced in excellent contributions by K. S. Rawji and V. W. Yong, H. Koennecke and I. Bechmann, D. Chatterjee et al., and T. Goldmann and M. Prinz, each illuminating slightly different aspects of microglial involvement and participation in demyelination and neurodegeneration. The controversial (i.e., unknown) roles that microglia may play in malignant glioma growth and invasion which continues to fascinate brain tumor biologists and their views are represented in contributions by A. C. C. da Fonseca and B. Badie and by J. Wei et al. Finally, there are an update on microglial immunophenotypes in the context of neuropathic pain by K. Li et al. and a beautiful review on microglial activities in the leech, that well-known invertebrate which can regenerate its CNS from scratch with the help of microglia.

